# Update on tocilizumab in rheumatoid arthritis: a narrative review

**DOI:** 10.3389/fimmu.2025.1470488

**Published:** 2025-02-24

**Authors:** Simone Parisi, Maria Chiara Ditto, Francesco Ghellere, Salvatore Panaro, Francesca Piccione, Richard Borrelli, Enrico Fusaro

**Affiliations:** ^1^ Rheumatology Unit, Azienda Ospedaliera Universitaria Città della Salute e della Scienza di Torino, Turin, Italy; ^2^ Allergy and Clinical Immunology Unit, Ospedale Mauriziano, Turin, Italy

**Keywords:** rheumatoid arthritis (RA), tocilizumab (TCZ), efficacy and safety, randomized controlled trials (RCTs), real-world evidence (RWE), IL-6 receptor inhibitor

## Abstract

Rheumatoid arthritis (RA) is a chronic inflammatory disease characterized by joint pain, swelling, and stiffness, affecting approximately 1% of the adult population. Tocilizumab (TCZ), a monoclonal antibody targeting the IL-6 receptor, has emerged as an effective treatment for RA. This narrative review provides an update on TCZ’s efficacy and safety based on data from randomized controlled trials (RCTs) and real-world evidence (RWE). TCZ, available in subcutaneous (SC) and intravenous (IV) formulations, has shown significant benefits in RA management. Key clinical trials, including SAMURAI, OPTION, RADIATE, and TOWARD, have demonstrated TCZ’s efficacy as monotherapy and in combination with conventional synthetic disease-modifying antirheumatic drugs (csDMARDs), particularly in patients with inadequate responses to methotrexate or TNF inhibitors. Long-term studies, such as STREAM, have highlighted TCZ’s sustained efficacy and favorable safety profile over 5 years. The impact of TCZ on cardiovascular health, lipid profiles, and the risk of infections has been a focal point, with findings suggesting no significant increase in cardiovascular disease risk compared to other RA therapies. RWE further highlights the effectiveness of TCZ, identifying predictors of response, such as age, and emphasizes its suitability for biologic-naïve and overweight patients. Special considerations include TCZ use in RA-associated interstitial lung disease and amyloidosis. Overall, TCZ remains a pivotal option in RA treatment, with a well-established safety and efficacy profile supported by extensive clinical and real-world data.

## 1 Introduction

Rheumatoid arthritis (RA) is a chronic inflammatory disease characterized by pain and swelling in the joints, along with stiffness and fatigue. This leads to enduring synovitis and gradual joint deterioration, resulting in reduced functionality and an elevated risk of illness and mortality. RA affects 1% of the adult population and stands as a significant contributor to disability (1). The global prevalence in Italy is estimated at around 0.5% (2). The pathogenesis of RA remains not fully understood. Individuals genetically predisposed to this condition develop it through interactions with various environmental factors, such as smoking habits (3). Moreover, the presence of the “shared epitope” is another significant genetic factor in RA predisposition, particularly for ACPA-positive RA (4).

Interleukin-6 (IL-6) plays a pivotal role in RA pathogenesis. It is a versatile cytokine with diverse roles in immunity, exhibiting both pro-inflammatory and anti-inflammatory effects. IL-6 is produced mainly by myeloid cells, and its dysregulation is linked to autoimmune diseases like RA. High levels of this cytokine are associated with RA disease activity, highlighting its significance in rheumatic conditions and inflammation. Depending on different types of stimuli, other cytokines, such as TNF-alpha and IL-1, stimulate the production of IL-6, triggering a series of reactions in both innate and adaptive immunity (3, 5–7). In terms of innate immunity, IL-6 plays a role in the maturation of inflammatory infiltrate by promoting neutrophil migration and mononuclear cell infiltration. Additionally, it acts as a chemoattractant for monocytes at the site of inflammation. Regarding acquired immunity, IL-6 exerts its effects on both T cells and B cells. Indeed, through T cells, it promotes the differentiation of B cells into active plasma cells leading to increased levels of serum gamma-globulins.

IL-6 exerts its effects through three different pathways: IL-6 signaling, IL-6 trans-signaling and IL-6 trans-presentation. In the first one, myeloid cells produce IL-6 in response to immune stimuli, which binds to IL-6R on target cells. This forms a complex with gp130, activating signaling pathways that induce acute phase protein production like C-reactive protein (CRP) in the hepatocytes (3, 8). In the second one, IL-6 binds to the soluble IL-6 receptor (sIL-6R) in the bloodstream, forming a complex that interacts with gp130 on various cell types, including those lacking membrane-bound IL-6 receptors. This enables broader cellular effects, signaling emergent events, such as an infection throughout the body (8, 9). The third signaling pathway is characterized by a unique mechanism where T cells respond to IL-6 despite lacking IL-6Rα expression. Dendritic cells present IL-6/IL-6Rα complex to T cells via gp130 molecules, distinct from traditional IL-6 pathways. Unlike other IL-6 signaling modes, IL-6 antibodies fail to inhibit trans-presentation, but anti-IL-6Rα antibodies can neutralize it (10).

Persistent dysregulation of IL-6 is linked not only with autoimmune diseases but also in some cancers since elevated IL-6 levels are involved in inflammation-driven tumors. In the elderly, a common pro-inflammatory pathway involving cytokines, such as IL-6 connects age-related conditions and promotes tumorigenesis (6, 11, 12).

Understanding IL-6 biology is crucial for IL-6-targeted therapies. In the context of RA, two classes of IL-6-targeted inhibitors are particularly relevant: anti-IL-6 receptor monoclonal antibodies, such as tocilizumab (TCZ; approved for RA in 2010), sarilumab (2017) and olokizumab (currently under investigation for RA), and anti-IL-6 monoclonal antibodies, such as siltuximab (2014) (7). However, it should be noted that siltuximab is approved for non-RA indications, such as Castleman disease, and is not intended for RA treatment. Targeting the IL-6 receptor, as seen with TCZ and sarilumab, offers advantages by potentially blocking other cytokines in the IL-6 family (3). The R4RA trial by Humby et al. demonstrated that stratification of patients based on synovial RNA sequencing improves the predictability of response to anti-IL-6 therapies (13). For instance, in B-cell-poor patients, tocilizumab showed superior efficacy compared to rituximab, highlighting the importance of tissue-specific molecular profiling in guiding treatment choices and advancing precision medicine (13, 14).

Anti-IL-6 therapies differ in their targets and applications. IL-6 receptor inhibitors like tocilizumab and sarilumab block both classic and trans-signaling, making them effective in RA. In contrast, ligand inhibitors such as ziltivekimab, under investigation for cardiovascular and renal diseases, focus on suppressing IL-6-driven inflammation in conditions like atherosclerosis. Ridker et al. highlight that ligand inhibitors might offer advantages in diseases where trans-signaling plays a key role (15). These differences are critical for tailoring treatments, with receptor inhibitors broadly impacting IL-6 activity while ligand inhibitors target specific inflammatory pathways (15).

**Figure 1:**
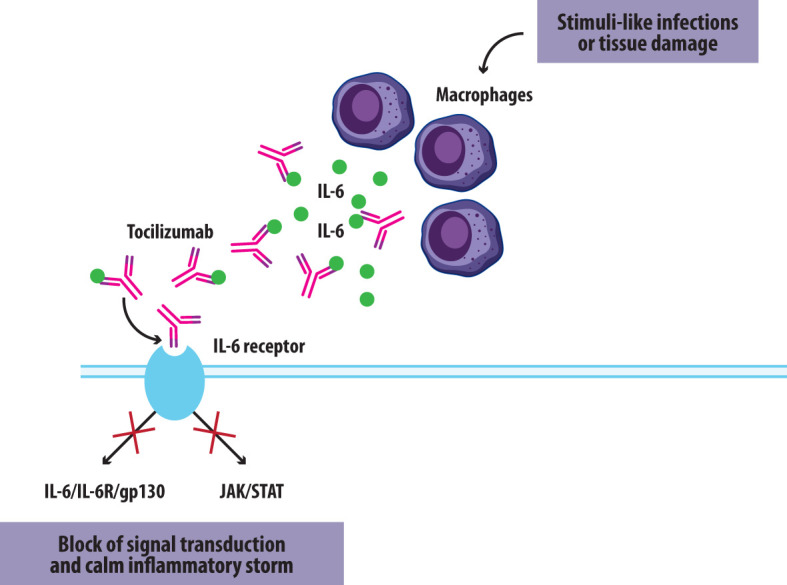
Tocilizumab mechanism of action.

TCZ, an approved therapy for RA, blocks both classic and trans-signaling pathways (**Figure 1**). Its success in RA treatment underscores IL-6’s significance, motivating the exploration of novel therapeutic avenues (9, 10). TCZ is a genetically engineered humanized monoclonal antibody created by grafting the complementarity-determining region of a mouse anti-human IL-6 receptor onto human IgG. It can dissociate the complex composed of IL-6 and soluble IL-6 receptor (sIL-6R), inhibiting both the classic pathway and the trans-signaling pathway, the latter constituting the pro-inflammatory activity of IL-6 (16). TCZ is administered either as an IV infusion or subcutaneous injection. TCZ was first approved in Japan for moderate to severe RA (2005), then in 2009 in Europe and in 2010 in the USA (**Table 1**). Currently, it is also being investigated for other conditions, including cytokine release syndrome and severe COVID-19, thanks to its significant anti-inflammatory properties. Thus, guidelines for the treatment of COVID-19 have included it for both severe forms and for children under emergency use (16, 17). As of 2022, the FDA approved TCZ for the treatment of COVID-19 in hospitalized adult patients receiving systemic corticosteroids and requiring oxygen support, as recommended in COVID-19 guidelines (18). Then, it was approved for emergency use in the treatment of COVID-19 pediatric patients aged 2 years to <18 years (19, 20).

**Table 1:** Summary of Tocilizumab market launch and label indications.

Year of market introduction	Brand name	Available formulations	Warnings and safety precautions	Year and approved indications for EU use
Japan 2005 EU 2009 USA 2010	Tosymra (Japan) RoActemra (EU) Actemra (USA)	Subcutaneous and intravenous	Risk of infections, infusion reactions, allergic reactions, gastrointestinal reactions, liver impairment, and reactions to concomitant medications. Recommend hematological monitoring	2009 Rheumatoid Arthritis 2011 Systemic Juvenile Idiopathic Arthritis 2017 Giant Cell Arteritis

When using TCZ, its impact on lipid profiles and its immunosuppressive effects must be taken into account, as they increase the risk of infections. Indeed, the 5-year extension STREAM study demonstrates that the drug maintains sustained long-term efficacy with a favorable safety profile, even if the rate of serious infections reported in 17.5% of patients enrolled in this study was 5.7 events per 100 patient-years (16). Moreover, regarding the trend toward a worsening lipid profile during TCZ treatment, another study demonstrated no statistically significant changes in it observed over the long term (21).

For autoimmune conditions, such as RA, beyond TCZ, sarilumab is also available. They are both IL-6 receptor inhibitors and have shown efficacy in RA monotherapy, demonstrating more efficacy than adalimumab (3). Differences between them include their structure, administration, dosage, and indications. TCZ is a humanized monoclonal antibody administered intravenously or subcutaneously, while sarilumab is fully human and given subcutaneously. Dosage frequency varies: subcutaneous (SC) TCZ is a 162 mg weekly injection, while intravenous (IV) formulation is given at the dosage of 8 mg/kg every 4 weeks; sarilumab SB is a 200 mg every 2 weeks injection. TCZ IV formulation may allow for dosage adjustment up to 4 mg/kg; sarilumab may allow for dosage reduction to 150 mg every 2 weeks. TCZ is approved for RA, juvenile idiopathic arthritis (JIA), systemic JIA, giant cell arteritis (GCA) and COVID-19, while sarilumab is indicated for moderate to severe RA. Biosimilar development reflects their established efficacy and safety (3, 10, 22–25). Biosimilar TCZ has demonstrated an efficacy and safety profile equivalent to that of the originator (26).

This narrative review aims to provide an update on TCZ in RA based on published data, randomized control trials, and real-world evidence.

## 2 Methods

To explore the literature about the use of TCZ in the management of RA, a PubMed search for full-text articles was conducted using the following search string: (((tocilizumab [Title/Abstract]) OR tocilizumab [Title/Abstract]) AND rheumatoid arthritis [Title/Abstract]). Inclusion criteria encompassed publications in the English language, for which abstracts were available. PubMed and EMBASE databases were searched for studies published between 1 January 2005 and 31 December 2023. The keywords for the search were: “tocilizumab”, “IL-6”, “IL-6 receptor”, “IL-6 inhibitor”, and “rheumatoid arthritis”. According to their related Emtree and Mesh terms, each database was searched with a specific string developed on these keywords.

## 3 Results

### 3.1 TCZ efficacy and safety profile emerging from randomized controlled trials

**Figure 2:**
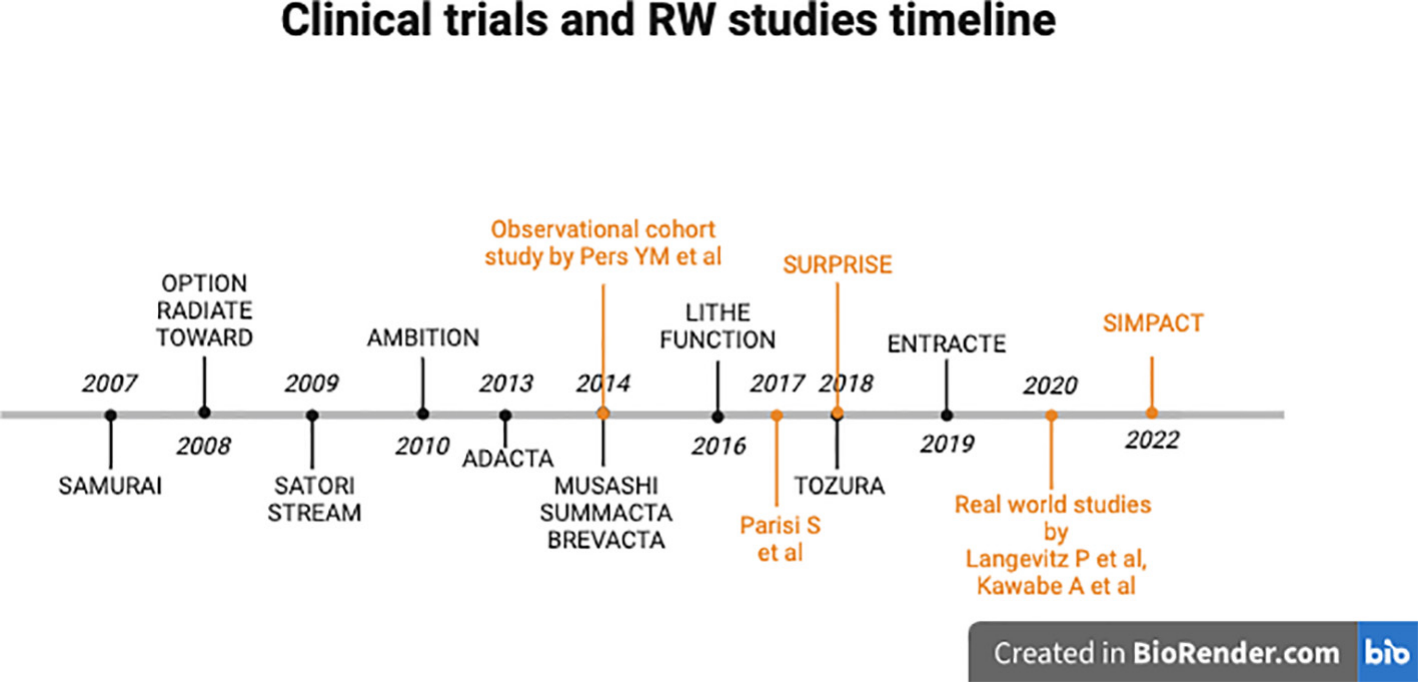
Timeline of clinical trials (black) and real-world studies (orange) involving Tocilizumab in RA.

Given the assumption that comparing results across clinical trials due to disparate patient populations with varying prior treatments and disease histories is challenging, below, we provide an overview of all clinical trials involving TCZ in the treatment of RA over the past 18 years (**Figure 2**, **Table 2**).

**Table 2:** Summary of registration studies.

Trial	Phase	Patients (n)	Design	Comparison group	Duration	Endpoint	Ref
SAMURAI	III	306	Open	csDMARDs	12 months	Inhibition of structural joint damage progression (TCZ monotherapy)	(27)
OPTION	III	623	Double-blind	MTX	6 months	ACR20 response at week 24	(28)
RADIATE	III	499	Randomized, double-blind, placebo-controlled	MTX	6 months	ACR20 response at week 24	(29)
TOWARD	III	1220	Double-blind	csDMARDs	6 months	ACR20 response at week 24; improvement of ACR50/70 at week 24	(30)
SATORI	III	127	Double-blind	MTX	6 months	ACR20 response at week 24	(12)
STREAM	III	143	Open-label	–	5 years	ACR improvement criteria, DAS28, and EULAR response	(12)
AMBITION	III	286	Randomized controlled	–	5 years	Long-term efficacy and safety up to 264 weeks	(31)
ADACTA	IV	452	Randomized, double-blind, parallel group	Adalimumab	6 months	Change in disease activity score using 28 joints (DAS28) from baseline to week 24	(32)
MUSASHI	III	348	Double-blind, parallel group, double-dummy, comparative	TCZ SB vs TCZ IV	6 months	Comparison of TCZ SC vs TCZ IV; ACR20 at week 24	(33)
SUMMACTA	III	1262	Randomized, double-blind, parallel group	TCZ SB vs TCZ IV?	2 years	ACR20/50/70 response at week 24 in TCZ SC vs TCZ IV group. Remission as DAS28 <2.6 and a decrease from baseline of ≥0.3 HAQ-DI at week 24	(34)
BREVACTA	III	656	Randomized, double-blind, placebo-controlled, parallel group	MTX	6 months	ACR20 week 24; radiographic progression and safety	(35)
LITHE	III	1196	Double-blind	MTX	1 year	Mean change from baseline in GmTSS and adjusted mean AUC for change from baseline in the HAQ-DI at week 104	(11)
FUNCTION	III	1157	Double-blind randomized controlled	MTX	6 months	Achieving remission (DAS28-ESR <2.6) at week 24 and radiographic efficacy by mTSS	(5)
TOZURA	IV	1804	Multinational, open-label, single-arm, common-framework	csDMARDs	6 months	DAS28-ESR at week 24. ACR response 20/50/70/90	(36)
ENTRACTE	IV	3080	Randomized, open-label, parallel group	Etanercept	5 years	ACR20 response week 24. Onset of MACE and related complications; all-cause mortality	(37)

csDMARDs, conventional synthetic disease-modifying anti-rheumatic drugs; MTX, methotrexate; SB, subcutaneous biologic; IV, intravenous.

Several phase III trials have demonstrated the clear efficacy of TCZ in various scenarios related to RA. In 2007, SAMURAI (in Japan) (27) and 2008 OPTION (28) evaluated the efficacy and safety of TCZ monotherapy compared to methotrexate monotherapy in patients with RA who were intolerant to methotrexate or had an inadequate response to it. RADIATE and TOWARD demonstrated the efficacy and safety of TCZ in patients with RA who had an inadequate response or intolerance to one or more TNF inhibitors (29) and also to conventional synthetic disease-modifying antirheumatic drugs (csDMARDs) (30). In 2009, STREAM was the first study to demonstrate the excellent long-term safety and efficacy of TCZ monotherapy over 5 years in patients with active RA. Not only were hemoglobin levels enhanced and the frequency of neutropenia reduced but also patients’ quality of life was strongly improved. Because an increase in total cholesterol levels is often associated with an elevated risk of cardiovascular disease, further investigation was needed to assess whether TCZ might contribute to an increased risk of developing ischemic heart disease (12). In the same year, the SATORI study demonstrated the efficacy and safety of TCZ in Japanese patients with RA who had an inadequate response to methotrexate (such as in other previous studies). In 2010, the AMBITION study established TCZ as an initial biological agent (monotherapy), demonstrating statistically superior clinical efficacy compared to a standard methotrexate (MTX) dose regimen (20 mg/week). While ACR20 was used as a regulatory endpoint, TCZ also achieved higher ACR50 and ACR70 response rates, which are more clinically relevant measures of meaningful improvement in RA. In this 6-month study, TCZ monotherapy exhibited greater efficacy in patients with relatively early active RA (according to the 2010 ACR/EULAR classification), for whom MTX had not previously failed, compared to MTX monotherapy. Besides, this study sustained that TCZ monotherapy causes lipid elevations and reversible neutropenia linked to IL-6R inhibition. The long-term significance of these effects is yet to be determined (31). In 2013, the ADACTA study compared the efficacy of TCZ monotherapy with adalimumab monotherapy in patients with RA who were intolerant to MTX or for whom continued treatment with MTX was considered inappropriate (32). In 2014, the MUSASHI study provided a sustained favorable safety and efficacy profile of TCZ as monotherapy in a Japanese cohort of RA patients. The study compared the subcutaneous formulation to the intravenous one, demonstrating its noninferiority. The availability of a subcutaneous formulation of TCZ offers a significant enhancement to the quality of life for RA patients due to shorter administration time and home administration. From week 24 to 108, there was a gradual increase in the proportion of patients who achieved a positive response and an improvement in the clinical response. Overall, after 108 weeks of exposure, there was no attenuation of the therapeutic response (33). In the same year, the SUMMACTA study compared the efficacy and safety of SC versus IV formulations of TCZ (TCZ SC 162 mg weekly versus TCZ IV 8 mg/kg every 4 weeks) in patients with RA with an inadequate response to biologic DMARD (bDMARDs). TCZ SC demonstrated higher efficacy in terms of ACR20 response, while the DAS28 remission was similar between the TCZ SC and the TCZ IV. Clinical safety profiles were comparable, except for a higher incidence of Injection Site Reactions more commonly seen with TCZ SC (34).

In 2014, the BREVACTA study aimed to assess the efficacy and safety of TCZ SC compared to subcutaneous placebo (PBO-SC) in patients with moderate to severe RA who had an inadequate response to bDMARDs. Notably, joint damages were reduced, and the incidence of infections and serious infections was similar between the treatment groups (35).

In 2016, LITHE investigated the efficacy, also radiologically, of TCZ in RA refractory to MTX patients. Radiological disease progression (according to the sharp total score) was reduced after two years of treatment (11).

In the same year, the FUNCTION study investigated the impact of inhibiting IL-6 signaling with TCZ as a first-line therapeutic option for RA in a population exclusively comprising MTX-naive patients with early progressive RA (5). Throughout the 52-week study, the group receiving 8 mg/kg TCZ in combination with MTX consistently demonstrated superior outcomes across all efficacy measures. This included improvements in clinical outcomes and enhanced functional ability (measured by HAQ-DI score). Additionally, the combination therapy inhibited joint damage progression, as evidenced by radiographic measures such as the van der Heijde-modified Sharp score, and achieved better disease control, as reflected by DAS28-ESR scores. While 8 mg/kg TCZ with MTX emerged as the most effective treatment, both 4 mg/kg TCZ with MTX and 8 mg/kg TCZ monotherapy proved to be good alternative treatments. These alternatives are particularly valuable for subsets of patients, such as those unable to tolerate MTX or the higher 8 mg/kg dose due to contraindications or adverse reactions (5).

In 2018, the TOZURA study evaluated the efficacy and safety of TCZ-SC as monotherapy or in combination with csDMARDs in patients with moderate to severe RA who had an inadequate response to csDMARD or anti-TNF agent therapy or who were MTX naïve. Results have demonstrated that TCZ-SC was efficacious in patients with RA, with combination therapy and monotherapy being comparably effective and with the observed safety profile being consistent with the known TCZ profile (36).

In 2019, the ENTRACTE trial compared the risk of major adverse cardiovascular events (MACE) in patients with RA treated with TCZ or the TNF inhibitor etanercept. Similar to findings from the STREAM study, given that RA is associated with a higher burden of atherosclerosis and increased mortality from atherosclerotic events and cardiovascular disease (CVD) compared to individuals without RA, the elevation of lipids with atherogenic potential raised concerns regarding the CVD risk-to-benefit ratio of TCZ in RA. However, the findings from this trial suggest that the risk of CVD following treatment with TCZ does not appear to be significantly higher than with etanercept, at least within the initial years of therapy (37).

The results of the ENTRACTE trial, summarized in **Table 3**, provide a detailed comparison of the cardiovascular safety outcomes between TCZ and etanercept. Notably, while the overall risk of MACE was comparable, specific differences were observed in adverse event profiles, including rates of serious infections and gastrointestinal perforations, highlighting the need for vigilant monitoring during TCZ therapy.

**Table 3:** Summary of ENTRACTE trial results.

Parameter	Tocilizumab group (number of events)	Etanercept group (number of events)	Hazard ratio (95% CI)	Comments
Major adverse cardiovascular events	83	78	1.05 (0.77–1.43)	No significant difference in MACE risk between groups
Cardiovascular-related death	36	35	1.03 (0.64–1.63)	Similar risk between groups.
Nonfatal myocardial infarction	28	31	0.89 (0.54–1.49)	Lower but nonsignificant trend of MI in tocilizumab group.
Nonfatal stroke (all types)	24	15	1.53 (0.80–2.92)	Higher incidence in tocilizumab group but nonsignificant.
Hospitalized heart failure	12	8	1.50 (0.61–3.67)	Small number of events with nonsignificant differences.
Adverse events related to infections	159 serious infections	111 serious infections	1.39 (1.08–1.79)	Infection-related AEs were more frequent in the tocilizumab group.
Gastrointestinal perforations	8	1	8.43 (1.06–67.26)	Significantly higher risk of gastrointestinal perforations in tocilizumab group.

About the intricate relationship between lipid metabolism and CV risk in RA, the research showed that RA patients often exhibit lower levels of traditional blood lipids, such as total cholesterol (TC), low-density lipoprotein cholesterol (LDL-c), and high-density lipoprotein cholesterol (HDL-c), particularly under hyperinflammatory conditions. This phenomenon is termed the ‘lipid paradox’ because, despite these lower lipid levels, RA patients have a significantly increased risk of CVD. The systemic inflammation characteristic of RA leads to alterations in lipid metabolism, resulting in dysfunctional HDL that promotes LDL oxidation and plaque formation. These changes in lipid subcomponents and their functions contribute to the increased cardiovascular risk observed in RA patients (38).

#### 3.1.1 TCZ’s broader implications or cardiovascular benefits

Emerging evidence suggests that TCZ may exert significant cardiovascular benefits beyond its established role in managing RA. By targeting IL-6, TCZ not only reduces systemic inflammation but also influences key mediators of cardiovascular risk, such as endothelial dysfunction, monocyte activity, neutrophil extracellular trap (NET) formation (NETosis), and oxidative stress – principal drivers of atherosclerosis and CVD. Ruiz-Limón et al. demonstrated that TCZ improved endothelial function, as assessed by postocclusive hyperemia using Laser Doppler, and decreased oxidative stress in monocytes and neutrophils from RA patients (39). TCZ also reduced the percentage of low-density granulocytes and inhibited NETosis generation, a known contributor to vascular damage and thrombosis. Furthermore, Ruiz-Limón et al. showed that TCZ reversed the pro-inflammatory and prothrombotic status of RA monocytes by modulating specific intracellular pathways (39).

These findings suggest that TCZ’s cardiovascular benefits extend beyond its anti-inflammatory properties to include direct vascular and cellular effects. Additionally, earlier studies by Kume et al. highlighted that TCZ attenuates arterial stiffness, as measured by the cardio-ankle vascular index (CAVI) and aortic augmentation index, further supporting its role in improving vascular health. The modulation of lipid profiles by TCZ, particularly its impact on increasing HDL cholesterol, adds another layer of potential cardiovascular benefit. By targeting IL-6, a cytokine implicated in atherogenesis and plaque destabilization, TCZ may positively influence atherosclerotic plaque progression and stability (40).

Taken together, these findings highlight TCZ’s potential to reduce the pro-atherothrombotic profile in RA patients through the restoration of endothelial function, inhibition of oxidative stress and modulation of monocyte and neutrophil activity (39). Its ability to attenuate arterial stiffness and improve lipid profiles further supports its promise. These properties position TCZ as a promising candidate for broader exploration in populations at high cardiovascular risk, such as those with subclinical atherosclerosis or metabolic syndrome (40).

### 3.2 TCZ efficacy and safety profile emerging from real-world studies

Results of several real-world studies that have evaluated the efficacy of TCZ and the safety of treatments in routine clinical practice have been analyzed (**Figure 2**, **Table 4**). Patients involved in these studies may significantly differ from those included in clinical trials. The RWE insights are valuable as they demonstrate the true impact of treatments in real-life settings.

**Table 4:** Summary of real-world studies.

Trial	Patients	Design	Comparison group	Duration	Endpoint
Pers et al. (41)	204	RW	–	6 months	Identify predictors of response and remission to TCZ in RA patients seen in daily routine clinical practice
Parisi et al. (42)	29	RW	–	6 months	Evaluate the response of TCZ in RA patients who are not responders to previous biologic therapy
Kaneko et al. (43) SURPRISE	105	RW	–	2 years	TCZ-free remission and low disease-activity rates, functional outcome, and radiological outcomes were assessed with the modified total Sharp score (mTSS) and safety. The efficacy of reinstituted TCZ/MTX was also evaluated
Langevitz et al. (44)	100	Multi-center, open-label, single-arm	–	6 months	Proportion of patients achieving remission and LDA based on the CDAI after 24 weeks of treatment with SC TCZ. Change in SDAI up to 24 weeks; proportion of patients achieving SDAI remission and LDA after 24 weeks; change in Disease Activity Score with DAS28-ESR up to 24 weeks
Kawabe et al. (45)	1362	RW	Abatacept	3 years	Effectiveness and safety of bDMARDs
Nagy et al. (46) SIMPACT	337	Open-label, non-controlled, non-randomized, non-interventional study	–	6 months	Change in DAS28 and CDAI scores, the proportion of patients achieving remission in the whole population and in subgroups defined based on prior RA treatment history, and age, weight or biological sex *post hoc*

RW, real world; TCZ, tocilizumab; RA, rheumatoid arthritis; MTX, methotrexate; bDMARDs, biologic disease-modifying anti-rheumatic drugs; CDAI, Clinical Disease Activity Index; SC, subcutaneous; SDAI, Simplified Disease Activity Index; LDA, low disease activity; DAS28-ESR, Disease Activity Score with 28 Joint Count using Erythrocyte Sedimentation Rate.

A retrospective observational real-life study conducted across five academic centers in France assessed the efficacy of TCZ in combination with csDMARDs or biologic-naive patients based on the European League Against Rheumatism (EULAR) response criteria. The study specifically included patients with a history of arterial hypertension, ischemic heart disease, stroke or arteritis. However, three predictors of a better response to TCZ were identified: young age, high baseline CRP level, and no history of CVD. The findings suggest that offering TCZ to young patients without previous CVD and with a CRP level >10 mg/l leads to greater effectiveness and lower rates of primary failure. These identified predictors of response are valuable as they enable personalized treatment, allowing the selection of the most suitable biologic agent based on the individual patient’s profile. This approach not only improves medical cost-effectiveness but also reduces the number of non-responding patients (41). Moreover, these findings suggest that patients with comorbidities, specifically those with CVD, are more likely to discontinue treatment for their cardiovascular condition. This leads to reduced efficacy in managing RA due to poor adherence to the therapy rather than being an issue related to TCZ.

An RWE study also demonstrated TCZ effectiveness in treating inflammation in RA patients, both with clinical evaluation and with ultrasonography with rapid reduction of the power Doppler signal (42).

In 2018, the SURPRISE study demonstrated that TCZ led to remission in more than 90% of patients and that, after TCZ discontinuation, continued MTX therapy maintained low disease activity (43).

Another real-life setting phase IV study program recruited patients who were administered TCZ-SC on a weekly basis for a minimum of 24 weeks, either as monotherapy or in combination with a csDMARD. The results align with findings from other real-life studies and other randomized controlled studies confirming the safety, tolerability, and efficacy profile of the treatment (44).

The FIRST registry is a prospective observational cohort study designed to assess the long-term safety and effectiveness of biologic therapies, including TCZ, in patients with RA. It aims to gather real-world data on the use of these treatments in routine clinical practice and to monitor their outcomes over time, encompassing a follow-up period of up to 5 years. This RW study highlighted the growing proportion of elderly individuals, so the importance of tailoring therapeutic approaches for elderly RA patients, considering the increased prevalence of comorbidities, arises. Notably, the FIRST study prioritizes the examination of pre-existing lung diseases among the various comorbidities. Elderly RA patients frequently exhibit heightened disease activity and more substantial functional limitations compared to their younger counterparts. Within the FIRST registry, findings indicate that the optimal effectiveness and safety of TCZ are observed in patients aged below 75 years. For patients aged 75 years or older, TCZ and abatacept (ABA) therapies may be considered suitable options. Additionally, in patients under the age of 65 years, TNF inhibitors demonstrated greater efficacy in improving disease activity, and they were associated with increased frequency of discontinuation due to remission. Thus, tailoring therapeutic strategies based on age groups emerges as a potential avenue to enhance the outcomes of bDMARD therapy for RA, addressing the unique considerations associated with age and comorbidities in this patient population (45).

The SIMPACT study aimed to assess the efficacy and safety of MTX-free TCZ-SC therapy in RA patients in a real-world setting. The study observed patients for a 24-week treatment period in Hungarian centers, where treating physicians prescribed TCZ-SC. The results indicated a significant reduction in disease activity measured by both DAS28 and CDAI, with a more pronounced clinical response observed in biologic-naïve patients and a lower response noted in patients over 75 years of age. While real-world clinical data on TCZ therapy in elderly patients is limited, recent findings align with those of the REACTION study, suggesting that younger age is associated with a better clinical response and remission rate 6 months after TCZ initiation (46). Additionally, the study reported a significant decrease in the frequency of co-administered medications, including oral corticosteroids (CSs) and DMARDs. To enhance the efficacy of bDMARDs, both EULAR recommendations and American College of Rheumatology (ACR) guidelines suggest supplementing bDMARDs with csDMARDs, such as MTX (46).

### 3.3 Tocilizumab efficacy and safety on specific populations

#### 3.3.1 Interstitial lung disease

Interstitial lung disease (ILD) encompasses a spectrum of disorders affecting the lung interstitium, including Usual Interstitial Pneumonia (UIP) and Non-Specific Interstitial Pneumonia (NSIP) patterns. UIP is characterized by fibrosis with honeycombing and traction bronchiectasis, often associated with a poor prognosis and commonly seen in idiopathic pulmonary fibrosis (IPF). In contrast, NSIP presents with more uniform interstitial inflammation and fibrosis, exhibiting a better response to treatment and associated with various connective tissue diseases. Distinguishing between these patterns is crucial for appropriate management and prognostication in ILD patients (47).

ILD stands as a significant extra-articular manifestation of RA, impacting its morbidity and mortality rates. Pulmonary manifestations of RA typically manifest within the initial five years of the disease, with instances where they precede joint symptoms. Interstitial lung disease (ILD) is fortunately not a frequent complication (3.2–5.9%) but may also be currently underestimated (48–51).

ILD can be attributed to the chronic inflammatory processes inherent to RA itself, as well as to the immunomodulatory effects of DMARDs used in its treatment. Some csDMARDs and bDMARDs have been linked to the onset or exacerbation of ILD, presenting difficulties in determining an appropriate and safe treatment strategy (48–50).

TCZ exhibits a favorable safety profile in patients with RA-associated interstitial lung disease (RA-ILD), potentially stabilizing lung involvement. Thus, while MTX has a limited role in ILD development and progression, TCZ monotherapy maintains efficacy, making it suitable for cases with both ILD and high articular disease activity, where MTX use is less recommended. Early ILD diagnosis in RA patients is crucial for understanding its natural history, identifying predictive factors, and evaluating the true impact of certain DMARDs, such as MTX, on this severe extra-articular complication (52, 53).

#### 3.3.2 Secondary amyloidosis

In secondary amyloidosis, hepatocytes produce the serum amyloid protein (AA), which forms insoluble extracellular deposits. Kidneys are the most commonly affected organs (>90%) (54), but amyloid deposition can also occur in other organs, such as the spleen, liver, heart, adrenal glands, thyroid glands, lungs and gastrointestinal tract. Systemic AA amyloidosis may arise from poorly controlled RA or in patients with a long history of RA, where pro-inflammatory cytokines, such as IL-6, play a key role in driving systemic inflammation. RA accounts for over 60% of cases of AA amyloidosis, whereas only 7–26% of RA patients develop amyloidosis (55). Since the first reports of TCZ for systemic AA amyloidosis emerged in 2006, when Okuda et al. reported improvements in serum AA amyloid levels, reductions in proteinuria, and histological improvement in a 26-year-old woman with juvenile idiopathic arthritis, there have been several reports confirming its efficacy (56, 57). TCZ decreased proteinuria and stabilized kidney function, thereby improving clinical disease activity. Furthermore, TCZ has shown benefits in treating AA amyloidosis associated with other various underlying conditions, including familial Mediterranean fever, multicentric Castleman disease, viral hepatitis (58) and amyloid heart disease (59). Additionally, the literature suggests that TCZ may preserve renal function even in cases of end-stage kidney disease, potentially delaying the progression of renal dysfunction in RA patients with AA amyloidosis (59). Interestingly, in two retrospective studies, it has been demonstrated that TCZ was more clinically beneficial (according to the DAS28 score) than anti-TNF therapy in patients with AA amyloidosis complicating rheumatic diseases (60, 61).

The mechanism underlying the efficacy of TCZ in AA amyloidosis has yet to be understood and may vary depending on the inflammatory status of the patient at treatment onset. It is hypothesized that TCZ is able to block the transcription of AA amyloid protein but also allows for regression of deposits already present, which may account for the improved GFR in some cases (62).

#### 3.3.3 Overweight/obesity

Obesity is considered a mild chronic inflammatory disease and has been identified as a risk factor for developing RA. White adipose tissue produces cytokines, such as TNF and IL-6, which have pro-inflammatory activity and are implicated in RA pathogenesis (63). Obesity has been reported to negatively impact the efficacy of cytokine-targeted therapies but not cell-targeted therapies, and this effect is more pronounced in women than in men (63).

Even though the impact of obesity on the effectiveness of TCZ in RA remains controversial (63), the response to this drug is not significantly affected by weight or BMI, contrary to other biologic therapies, such as TNF inhibitors, making it a viable treatment option for overweight or obese RA patients (64, 65).

In normal weight individuals, adipose tissue is composed of adipocytes that cooperate with immune system cells, which secrete molecules contributing to the maintenance of an anti-inflammatory phenotype. As body weight increases, adipocytes enlarge and produce chemotactic molecules that recruit immune cells, primarily monocytes, from the circulation. These monocytes infiltrate the adipose tissue, differentiating into a pro-inflammatory state. Once differentiated, macrophages secrete pro-inflammatory cytokines, such as IL-6 and TNF, which act in both autocrine and paracrine manners with adipocytes, perpetuating the inflammatory state within the tissue. These cytokines are then also released into the circulation, promoting the systemic inflammation characteristic of obese individuals (66). This is documented by the direct correlation between increased BMI and circulating levels of the two cytokines. It is interesting to note that in obese patients, adipose tissue contributes to approximately 30% of the circulating levels of IL-6 (67). Furthermore, by improving RA control and enabling greater physical activity, biologics like TCZ may indirectly aid in weight management and the associated inflammatory burden.

#### 3.3.4 Pregnancy and breastfeeding

Initial recommendations from the European Medicines Agency (EMA) sustained that TCZ should not be used during pregnancy unless absolutely necessary. Women of childbearing age should use effective contraception during treatment with TCZ and for up to 3 months after discontinuation. Currently, TCZ has been approved and advised for women who are pregnant or may become pregnant only when the potential benefits of treatment outweigh the potential risks. Even though there are many documented cases in which TCZ was successfully used throughout pregnancy with no abnormalities recorded in the newborns, effective contraception is strongly recommended due to limited data on its safety (68, 69). It is important to note that better control of RA during pregnancy, even with the use of biologics, such as TCZ, is generally more beneficial for both the mother and the baby than the risks associated with uncontrolled disease.

About breastfeeding, the passage of TCZ into breast milk is currently unknown. Saito et al. showed that TCZ might be safe for both pregnancy and breastfeeding because of its low degree of transplacental transmission. However, since information is still limited, the indications for TCZ should be carefully considered, and its use should be approached with caution in pregnant women and during breastfeeding (70).

#### 3.3.5 Elderly patients

Data emerging from a large observational study (ICHIBAN), which involved elderly patients (>65 years old), show that long-term TCZ treatment is effective and has an acceptable safety profile compared to younger patients (71). In real-world conditions in Germany, patients with RA treated with TCZ for up to 2 years generally did not discontinue therapy due to adverse events, except for cases involving elderly patients who experienced infections (71).

Patients with age-associated comorbidities (such as diabetes, coronary heart disease, anemia, renal impairment, lung disease, infections and malignant tumors) treated with TCZ experienced reductions in RA disease activity compared to those without such comorbidities (72). When administering a biologic drug concomitantly with MTX to treat this kind of population, achieving an adequate MTX dose may be challenging, leading to decreased efficacy. Prolonged steroid therapy should be used cautiously because it can induce progressive osteoporosis and increase the risk of fractures in elderly patients (73). As demonstrated by Bauer in 2020 (74), immunosenescence in elderly patients with RA is characterized by reduced thymic output and expansion of senescent T cells, leading to a compromised immune system. Senescent T cells, particularly the CD28- subset, exhibit a pro-inflammatory phenotype known as the senescence-associated secretory phenotype (SASP), which contributes to chronic inflammation and disease progression. This cascade of events exacerbates RA by promoting the release of pro-inflammatory cytokines, such as IL-6 (74), thereby further supporting the usage of TCZ in these patients.

### 3.4 Tocilizumab adverse effects and general considerations

The most frequent adverse effects reported in the literature after TCZ treatment are infections, neutropenia, malignancies and diverticulitis.

#### 3.4.1 Infections

Infections, particularly those involving the respiratory and urinary tract, could arise as the result of the inhibition of the IL-6 pathway, which compromises the host’s defense against various microorganisms. The TOWARD study presents data on serious infections, complications of diverticulitis, hypersensitivity reactions and tuberculosis reactivation. Other infections include invasive pulmonary infections such as candidiasis, aspergillosis, coccidioidomycosis, pneumocystis jirovecii pneumonia, cellulitis, herpes zoster, gastroenteritis, diverticulitis, sepsis, and bacterial arthritis (30). The RADIATE study showed the efficacy of TCZ plus MTX in patients with an inadequate response to TNF antagonist treatment, reported a case of staphylococcal polyarthritis infection after TCZ (in the 8 mg/kg group) and a case of necrotizing pneumonia (in the 4 mg/kg group), both of which resolved without sequelae. No cases of tuberculosis or opportunistic infections were observed (30). The STREAM study noted pneumonia, herpes zoster, and acute bronchitis as the most frequently reported infections. At least two patients with a history of tuberculosis received TCZ without experiencing recurrence or exacerbation of tuberculosis despite the lack of prophylactic use of antituberculosis drugs (12). However, the risk of TB can be effectively mitigated through adequate screening and management, which has made TB a minor concern in well-trained rheumatology practices. Compared to corticosteroids, commonly used in RA, TCZ offers a more targeted mechanism, avoiding the broad immunosuppressive effects of corticosteroids. Corticosteroids are linked to a higher risk of serious infections, including tuberculosis and fungal infections. While TCZ increases infection risk, it is comparable to or potentially lower than prolonged corticosteroid therapy, which also carries risks like impaired wound healing and metabolic complications.

A retrospective real-world study investigated the risk of HBV reactivation in patients undergoing long-term TCZ therapy for RA. The study highlighted the growing recognition of the risk of hepatitis B virus (HBV) reactivation during immunosuppressive therapy, including in rheumatology. Biological agents like TCZ, which decrease IL-6 levels therapeutically, may pose a risk of HBV reactivation since IL-6 inhibits HBV replication. Current treatment guidelines recommend initiating antiviral prophylaxis before immunosuppressive or cytotoxic therapy in HBsAg+ patients at high risk of HBV reactivation. Interestingly, none of the HBsAg+ patients who received antiviral prophylaxis experienced HBV reactivation in this study. Notably, HBV reactivation in HBsAg+ patients often occurred within the first year of TCZ treatment and could lead to fulminant hepatitis despite early preemptive treatment. Even HBsAg-/HBcAb+ patients, who have a very low risk, still require monitoring of HBV DNA and HBV markers to mitigate any potential reactivation risks (75). Adequate vaccination screening and updates are essential before initiating immunosuppressive therapies, such as TCZ. This includes assessing and addressing hepatitis B immunity to reduce the risk of HBV reactivation during treatment.

#### 3.4.2 Neutropenia

Neutropenia is another frequent adverse effect in patients receiving TCZ. Some possible mechanisms by which TCZ may result in lower neutrophil counts include blocking IL-6-induced neutrophil survival, downregulation of other inflammatory cytokines, and margination of neutrophils from the circulation into tissues. In the TOWARD study, during the double-blind controlled period and with long-term exposure, the pattern and incidence of decreases in neutrophil counts remained consistent with what was seen in the 6-month controlled clinical trials. Grade 3 neutropenia occurred in 3.7% of patients receiving TCZ and none of the patients in the control group, and no grade 4 neutropenia was reported. The transient nature of grade 3 neutropenia, the lack of association with infection in this 24-week study, and the lack of need to adjust concomitant treatment suggest that this effect is not a significant issue. However, evaluation of the impact of lower neutrophil counts during long-term treatment will require long-term follow-up (30). A reduction in mean neutrophil counts occurred also in the RADIATE study, albeit transiently (29). Prolonged neutropenia may increase the risk of serious infections in patients treated with TCZ. Nonetheless, the superior efficacy of TCZ provides initial evidence of a benefit–risk profile that supports its use in patients with active moderate to severe RA (31). Grade 2 neutropenia was observed in 17 patients and grade 3 in nine patients of the STREAM study; however, all events were transient, and no patients experienced neutropenia with fever or withdrew due to neutropenia (12).

#### 3.4.3 Malignancies

The LITHE study showed that malignancy rates were higher in the 4 mg/kg tocilizumab-MTX group (1.92/100 PY; total 521.90 PY) compared to the placebo-MTX (0.70/100 PY; total 284.81 PY) and 8 mg/kg tocilizumab-MTX (0.98/100 PY; total 1320.41 PY) groups. Twenty-three malignancies were reported in tocilizumab-treated patients up to week 104, with 17 of these reported within the first 52 weeks of the study. The most commonly reported malignancies were basal cell carcinoma (4 patients) and prostate cancer (two patients). All other malignancies were reported once (including cervix carcinoma, lung squamous cell carcinoma, endometrial cancer, gastroesophageal cancer, renal cell carcinoma, thyroid cancer, and others). Overall, malignancy rates were low among the tocilizumab- and placebo-treated groups and within the range observed in other populations of patients with RA. However, as observed during year 1 of LITHE, malignancy rates during year 2 remained higher in the 4 mg/kg tocilizumab-MTX group compared to the placebo-MTX and 8 mg/kg tocilizumab-MTX groups. The reason for the higher malignancy rate in the 4 mg/kg tocilizumab-MTX group during year 1 is unclear; however, the rate was unlikely to change significantly during Year 2. Data from large registries, including ARTIS, provide long-term evidence suggesting no increased malignancy risk in RA patients treated with biologics. For example, the study by Wadström et al. demonstrated that the risk of malignancies in biologic-treated patients was comparable to that of the general RA population, further supporting the overall safety profile of these therapies over extended periods (76). During year 2, the number of patient-years (PY) increased by 13% in the placebo-MTX group, 60% in the 4 mg/kg tocilizumab-MTX group, and 321% in the 8 mg/kg tocilizumab-MTX group, mainly because most patients switched from placebo-MTX or 4 mg/kg tocilizumab-MTX to 8 mg/kg tocilizumab-MTX in year 2. Increased malignancy rates were not observed in the 4 mg/kg tocilizumab groups of other phase III studies (77).

It should be emphasized that RA is associated with an increased risk of developing various types of cancers, as supported by multiple studies. This elevated cancer risk is partly due to the chronic inflammation and immune dysregulation inherent to RA. The study by Huss et al. reveals that patients with RA have a higher incidence of malignancies compared to the general population, with hazard ratios (HR) of 1.2, indicating a 20% increased risk overall (78). Hence, RA treatments, particularly with bDMARDs and targeted synthetic DMARDs (tsDMARDs), do not consistently increase the overall cancer risk, although certain drugs, such as abatacept, show a potential signal for increased cancer risk after prolonged treatment periods (78). Thus, ongoing surveillance and individualized risk assessment in RA patients undergoing such therapies, especially considering the complex interplay between the disease, its treatments, and cancer risk, is an unmet need (78).

#### 3.4.4 Intestinal injuries

Intestinal mucosal injury induced by TCZ is rare and typically occurs under specific circumstances. In patients receiving TCZ treatment, symptomatic diverticulitis was found to be more frequently associated with perforation compared to other treatments. Studies suggest that the risk of diverticular perforation may be slightly higher in patients treated with TCZ compared to csDMARDs or anti-TNF agents but lower than that associated with corticosteroids (79, 80). This type of mucosal injury often occurs in the presence of diverticulosis. The mechanism behind intestinal perforation involves TCZ, potentially masking abdominal pain and suppressing the elevation of CRP, thus impeding the healing process of intestinal injuries caused by diverticulitis. However, the exact pathological mechanism underlying TCZ-induced intestinal ulcers remains unclear. Cases of intestinal perforation as a complication of TCZ treatment have been linked to concomitant diverticulosis. There have also been reports of COVID-19 patients treated with TCZ developing ulcerative lesions that spread from the ileum to the ascending colon (81). Gastrointestinal perforation (GIP) represents another rare yet severe complication occasionally observed in RA patients. The risk of both GIP and diverticulitis appears to rise with TCZ therapy for RA. In susceptible RA patients, the neutralization of IL-6 may contribute to diverticulitis, potentially altering colonic contractions and leading to an unusual inflammatory presentation. Consequently, the gastrointestinal epithelium may fail to repair the initial lesion, potentially culminating in GIP (63).

## 4 Conclusions

Overall, results from completed clinical trials demonstrated that:

- TCZ efficacy and safety profile compared to other drugs, TNF inhibitors and other bDMARD (11, 29, 30, 36);
- TCZ has a safety and efficacy profile as monotherapy in patients with RA, available in two equivalent formulations (SC and IV) (33, 34);
- The risk of CVD following treatment with TCZ is not significantly higher when compared to the risk associated with other drugs, such as TNF inhibitors (12, 37);

Results from real-world findings add to the established body of knowledge and important evidence and strengthen previous findings from clinical trials and other real-world data. In particular, they demonstrated that:

- Smoking is not associated with a poorer response to TCZ (41);
- TCZ treatment shows an age-related decrease in efficacy and is more effective in biologic-naïve patients (46).

About TCZ efficacy and safety on specific populations, it has been demonstrated that:

- TCZ exhibits a favorable safety profile in RA-associated ILD, potentially stabilizing lung involvement (48);
- TCZ reduces AA amyloid deposition in various organs (56, 57);
- TCZ a suitable option for overweight or obese RA patients (65);
- Limited data on TCZ in pregnant and breastfeeding women TCZ safety warrants careful consideration of its use (68, 69);
- TCZ treatment in elderly patients with age-related comorbidities resulted in reduced RA disease activity (72).

The most frequent adverse effects of TCZ include serious infections, neutropenia and diverticulitis attributed to its mechanism of action. Understanding and monitoring these adverse effects are crucial for optimizing the safety and efficacy of TCZ therapy in RA patients (12, 29–31, 75, 79, 80).
